# Effect of Foot Reflexology and Benson Relaxation on Pain, Breastfeeding and Weight of Neonates of Women Undergoing a Cesarean Section

**DOI:** 10.34172/aim.2023.58

**Published:** 2023-07-01

**Authors:** Farnoosh Tavallali, Hakimeh Vahedparast, Fatemeh Hajinezhad, Razieh Bagherzadeh

**Affiliations:** ^1^Student Research Committee, Bushehr University of Medical Sciences, Bushehr, Iran; ^2^Department of Medical-Surgical Nursing, School of Nursing and Midwifery. Bushehr University of Medical Sciences, Bushehr, Iran; ^3^Department of Nursing, School of Nursing and Midwifery, Bushehr University of Medical Sciences. Bushehr, Iran; ^4^Department of Midwifery, School of Nursing and Midwifery, Bushehr University of Medical Sciences, Bushehr, Iran

**Keywords:** Breastfeeding, Benson relaxation, Complementary medicine, Pain, Reflexology

## Abstract

**Background:**

Given the negative effect of postoperative pharmacological pain control on breastfeeding, the present study aimed to compare the effects of reflexology and Benson relaxation methods on pain, breastfeeding, and infant weight gain in women undergoing a cesarean section (C/S).

**Methods:**

This randomized clinical trial was conducted on 135 women undergoing a C/S in the Gynecology Ward of Bushehr Persian Gulf Martyrs Hospital in Bushehr, Iran, in 2020. The participants were selected using convenience sampling, and randomly divided into three groups of foot reflexology, Benson relaxation, and control. The interventions were performed two hours post-operation and six hours after the first intervention. The pain score was measured and recorded immediately, and 30 and 60 minutes after each intervention. Breastfeeding frequency and duration were also assessed in the first 18 hours of birth. The infants’ weight was assessed at birth and 10 days later. Data analysis was performed using inferential statistics, chi-square or Fisher’s exact test, Kruskal-Wallis test, Mann-Whitney U or one-way ANOVA, Wilcoxon test and logistic regression analysis.

**Results:**

There was a larger decrease in the pain score of the reflexology and Benson relaxation (*P*<0.01) groups after the first and second interventions, compared to the control group. The breastfeeding frequency was higher in the two intervention groups, compared to the control group (*P*<0.001). Furthermore, the rate of return to the birth weight in ten days of birth was higher in the reflexology (*P*<0.01) and Benson groups (*P*<0.05) than the control group.

**Conclusion:**

Both the reflexology and the Benson relaxation methods effectively decreased pain and increased breastfeeding frequency and the infant’s weight gain.

## Introduction

 Breastfeeding has been recognized as the best method of nutrition in the first year of life.^1^ Early and repetitive feeding of the infant positively affects milk production and the duration of lactation. Pain, nausea, and instability of vital signs, as well as the medication used after a cesarean section (C/S) can increase the length of the patient’s recovery, which can delay mother-infant bonding and the breastfeeding process.^1-3^ Therefore, pain management is crucial for mother-infant bonding and breastfeeding initiation.

 Postoperative pain relief following a C/S can be provided by systemic administration of analgesic drugs, which are associated with maternal and neonatal complications.^2^ The use of opioids for pain management leads to decreased level of consciousness in the mother and the neonates, thus hindering effective maternal-neonatal relationship and inhibiting oxytocin secretion. Additionally, the morphine content prevents breast milk secretion.^4^ Accordingly, there is a need for non-pharmaceutical pain relief methods in this regard.^5^

 Complementary and alternative medicine is defined as treatments used in addition to or instead of the regular medical care.^6^ In this respect, reflexology is one of the most widely used complementary therapies for pain management.^7,8^ Reflexology, or zone therapy, involves the application of pressure to the reflex areas, with specific thumb, finger, and hand techniques. The reflex points on the feet and hands are thought to correspond to other areas of the body.^9^ Therefore, applying pressure on reflex points on the feet relieves pain in other body parts related to that point.^10^ To date, many studies have evaluated reflexology in various situations, most of them reporting its positive effect on pain relief.^5,11-16^ Nevertheless, few studies have focused on the effect of this complementary therapy on breastfeeding.^17-19^ In addition, no research has assessed the effect of reflexology on the infant’s weight gain.

 Saatsaz et al, Dhanalakshmi et al, and Çankaya & Ratwisch reported the positive effect of foot massage on breastfeeding.^2,17,18^ It is notable that reflexology requires an operator, and each session lasts half an hour. Therefore, comparing this technique with other non-pharmaceutical pain relief methods such as relaxation that can be performed by the patients themselves, could help with discovering user-oriented techniques. The relaxation technique is defined as a state of relative freedom from both anxiety and skeletal muscle tension.^20,21^ Studies have demonstrated the effect of this method on different pains.^5,22-24^

 A few studies have compared the effect of the relaxation method with reflexology. In a study by Mokhtari Noori et al, foot reflexology and relaxation effectively reduced pain in patients following a C/S; and reflexology had a higher impact on pain alleviation.^20^ Nonetheless, no study has assessed the impact of the Benson relaxation method on neonatal outcomes, including the number and duration of breastfeeding and the infant’s weight gain. Considering the above, two questions were raised in the present study: 1) Do foot reflexology and Benson relaxation methods affect the frequency and timing of breastfeeding, as well as the infant’s weight gain in the first 10 days of birth by reducing pain following a C/S? and 2) which method has a higher impact?

## Materials and Methods

###  Study Design 

 This randomized clinical trial aimed to compare the effects of the reflexology and the Benson relaxation methods on pain, breastfeeding, and the infant’s weight gain in women undergoing a C/S.

###  Participants

 The study was conducted on women undergoing a C/S in Bushehr Persian Gulf Martyrs Hospital (Iran) in 2020. With a total type I error of 0.05, a power of 80% and the mean and standard deviation of the pain score in intervention and control groups at 4.20 ± 1.27 and 5.66 ± 1.64 respectively based on a study by Mokhtary et al,^2,5^ a sample size of 28 subjects was calculated for each group. Given that this sample size is for comparing the two groups, it was modified using the sample size correction formula to compare more than two groups, which finally included 40 individuals for each group. After considering a 10% drop-out rate, 135 subjects were included in the study (45 subjects for each group). G power 3.1.9.2 was used for sample size calculation.

 The participants were selected using convenience sampling, and then randomly allocated to three groups of foot reflexology, Benson relaxation, and control. Block randomization was performed using 22 six-part and 1 three-part blocks.

###  Inclusion and Exclusion Criteria

 The inclusion criteria were non-emergency C/S, willingness to participate, spinal anesthesia, a minimum and maximum pregnancy age of 37 and 42 weeks, an age range of 18‒35 years, being aware of time and space, having no psychological disorders, first or second pregnancy, having a low-risk singleton pregnancy, having had no concomitant surgery (e.g. hysterectomy and tubectomy), good foot health (especially the sole), no cuts, burns, fungal infections, varicose veins, warts, corns, or diabetic neuropathy (numbness in the foot), having no sensitivity to touch or massage, no congenital anomalies detected on the ultrasound, having no pregnancy complications (e.g. hypertension), and no rupture of membranes. The exclusion criteria were severe complications during or after surgery (e.g. excessive bleeding, acute infection, etc), operating room incidents, need for more care in the intensive care unit, having had a non-transverse incision on the uterus or abdomen based on the patient’s operation description, pain score < 3 when deciding for the intervention, sensitivity to touch in the feet (in the foot reflexology group), neonatal mortality, severe abnormalities in the infant or infant hospitalization in the first 10 days of birth, and readmission of the mother.

###  Measures

 The data were collected using a demographic characteristics questionnaire, an infant status recording checklist, child weighing tools and a visual analog scale (VAS), which is a straight horizontal line with 10 columns. The straight line indicates lack of pain, and the smallest and largest columns demonstrate the lowest (one) and highest severity of pain. VAS is a single-item tool with approved reliability and validity.^26,27^ The infant status recording checklist includes the frequency of breastfeeding, neonatal birth weight, and the weight of a 10-day-old infant.

###  Intervention

 After receiving permission from the Vice-Chancellor for Research of Bushehr University of Medical Sciences and approvals from the Ethics Committee of the university, registration on the Iranian Registry of Clinical Trials, and acquiring permission from director of Persian Gulf Martyrs Hospital, the researcher visited the participants in the gynecology ward on the operation day. First, the research objectives were explained to the participants and informed consent was obtained prior to the study. They were then randomly allocated to three groups of foot reflexology, Benson relaxation, or control based on their designated blocks. Although blinding was not possible in this study, in order to reduce the exchange of information about the type of intervention between the three groups, the subjects in the intervention and control groups were placed in separate rooms. The demographic characteristics questionnaire was completed by all the participants before entering the operating room. Two hours post-surgery, the pain level was assessed and recorded, and the Benson relaxation or reflexology interventions were performed for the participants depending on their group. To attain reliable data, reflexology was carried out for all the participants by one of the researchers who had a relevant certificate and possessed the necessary theoretical and practical skills. Before the intervention, the researcher washed and dried her hands and massaged her hands with baby oil in order to properly warm them and make the process easier. First, a general massage was performed for two minutes to prepare the feet. Afterwards, the reflex points (pituitary gland, solar plexus, and uterine) were massaged for 10 minutes by applying constant or rotational pressure (20 minutes for both feet).^28,29,30^

 In the Benson relaxation group, the technique was taught to the patients as follows: (1) Sit quietly in a comfortable position. (2) Close your eyes. (3) Deeply relax all your muscles, beginning at your feet and progressing up to your face, and keep them relaxed. (4) Breathe through your nose, be aware of your breath, gently exhale through the mouth while repeating a calming word, and breathe normally. (5) Repeat the process for 20 minutes, try to completely loosen up; after 20 minutes, open your eyes slowly and remain in the same position for a few minutes. (6) Do not worry about whether you are successful in achieving a deep level of relaxation, and permit relaxation to occur at its own pace; when distracting thoughts occur, try to ignore them by not dwelling upon them.

 In order to simplify the process, prevent forgetfulness, and overcome the ambient noise, a flash memory containing instructions on the Benson relaxation method was provided to the participants in the Benson relaxation group. In the control group, routine care was performed to control pain, including one or two doses of intravenous pethidine and then administration of ketorolac or diclofenac suppository based on the patient’s complaint of pain.

 The second intervention was performed six hours later and exactly similar to the first one. Pain was measured and recorded immediately before, immediately after, and 30 and 60 minutes after the first and second interventions. In the control group, pain was recorded simultaneously with the two intervention groups. The mean pain score of the three episodes was considered as the pain score after each intervention. Upon the neonates’ entrance to the ward, the infant’s weight was measured using a Beurer scale (Germany) with a 5-g error rate. The frequency and duration of each breastfeeding session were also recorded after entering the gynecology ward up to 18 hours later. Ten days after the C/S, the neonates’ weights were measured and recorded by the researcher. The weight control location of 10-day neonates was chosen at the mother’s convenience and the previous arrangements. At the beginning of the study, the mother’s telephone number was taken to arrange for the infant’s weight control.

###  Statistical Analysis

**Figure 1:**
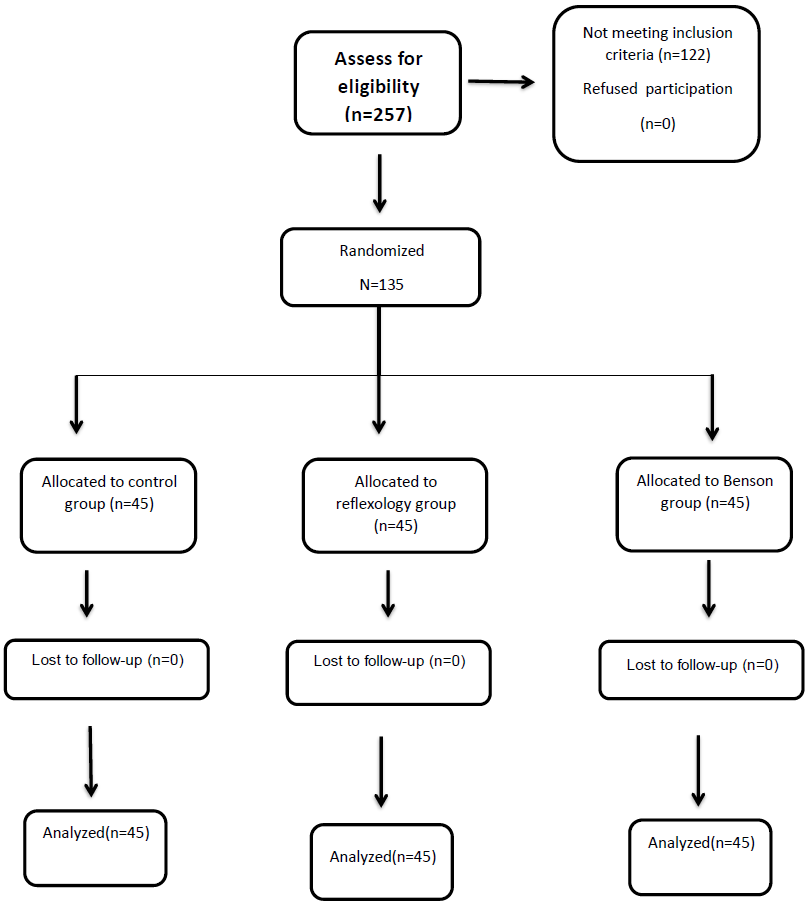
Consort Diagram

 At the beginning of the study, the intention-to-treat analysis was considered. Finally, all the participants in each group remained in their respective groups. Therefore, the intention-to-treat analysis was identical to the per-protocol (Figure 1). Data analysis was performed in SPSS 19 using descriptive (number, percentage, mean, and standard deviation) and inferential statistics. Chi-square or Fisher’s exact test were used to compare demographic variables and to compare the frequency of breastfeeding across the three groups. Wilcoxon test was used to make comparisons within groups of pain scores (pre-intervention and mean of immediately after, 30 and 60 minutes after the intervention as post-intervention) in each group. Kruskal-Wallis test (Mann-Whitney U as post hoc test) was used for comparing pain scores (pre-intervention, post-intervention and changes from pre- to post-intervention) as well as the duration of breastfeeding across the three groups. One-way ANOVA with Bonferroni test was used to compare birth weight and mean change between birth weight and the tenth-day weight across the three groups. In the end, logistic regression analysis was undertaken to examine the effect of the intervention on pain after the intervention (mean of immediately after, 30 and 60 minutes after the intervention pain score as post-intervention pain equal to or greater than median versus less than median) frequency of breastfeeding during 18 hours (more than median versus less than median) and return to the birth weight on the 10^th^ day of birth (return to the birth weight versus no return( by adjusting for confounding variables, which were related to the dependent variable in univariate logistic regression.

## Results

 In total, 257 women were initially considered to participate in the study. Of these, 122 did not meet the inclusion criteria. Finally, 135 women were divided into three groups. All the participants remained in their groups until the end of the study (Figure 1). There was no significant difference across the groups regarding their demographic variables (Table 1).

**Table 1:** Demographic Characteristics in the Three Groups

**Variable**	**Variable Level**	**Reflexology**		**Benson Relaxation**		**Control**	
		**No.**	**%**	**No.**	**%**	**No.**	**%**
Age	18‒24	10	22.2	4	8.9	6	13.3
	25‒35	35	77.8	41	91.1	39	86.7
Residential Status	Urban	26	57.8	28	62.2	24	53.3
	Rural	19	42.2	17	37.8	21	46.7
Education	Primary	4	8.9	1	2.2	3	6.7
	Secondary school	19	42.2	5	11.1	3	6.7
	Diploma	22	48.9	20	44.4	12	26.7
	Graduate	4	8.9	19	42.2	27	60.0
Employment	Housewife	41	91.1	38	84.4	34	75.6
	Employed	4	8.9	7	15.6	11	24.4
History of previous C/S	Yes	26	57.8	23	51.1	25	55.6
	No	19	42.2	22	48.9	20	44.4
Cause of C/S	Maternal request	31	68.9	27	60.0	33	73.3
	Medical or obstetric indication	14	31.1	18	40.0	12	26.7

C/S, Cesarean section. There were no differences between the three groups in terms of demographic variables (*P* value > 0.05).

 Based on the within-group comparison, the mean pain scores significantly decreased after the intervention in the reflexology and Benson relaxation groups (in both the first and second interventions). However, no significant difference was observed in the control group. In addition, a difference was detected among the three groups regarding the mean changes in pain score pre- and post-intervention (in both the first and second interventions) (Table 2). Moreover, a pairwise comparison revealed that the mean pain score further decreased in the first intervention in the reflexology group (*P*= 0.001) and in the Benson relaxation group (*P*= 0.003), compared to the control group. There was no significant difference between the Benson relaxation and the reflexology groups in terms of the decrease in the pain scores (*P*= 0.609). For the second round of the intervention, a pairwise comparison showed that the pain score further decreased in the second intervention in the reflexology (*P*< 0.001) and Benson relaxation (*P*< 0.001) groups, compared to the control group. The results were indicative of no significant difference between the Benson relaxation and the reflexology groups in terms of decreased pain post-intervention (*P*= 0.557).

**Table 2:** Within- and Between-Group Comparison of Pain Scores and Comparison of Mean Changes before and after the First and ‎Second Round of Intervention Across the Three Groups

**Pain Variable**	**Reflexology**		**Benson Relaxation**		**Control**		**(*****P *****Value) for Between Groups**
	**Mean±SD**	**Mean Rank**	**Mean±SD**	**Mean Rank**	**Mean±SD**	**Mean Rank**	
Pre intervention 1	6.91 ± 2.32	62.67	6.64 ± 1.99	57.24	8.16 ± 2.06	64.09	0.080
Post intervention 1	6.19 ± 2.22	57.64	5.99 ± 1.96	53.83	8.07 ± 1.60	92.52	< 0.001
*P *value for within groups	< 0.001		< 0.001		0.674		
Pre intervention 2	5.58 ± 1.94	66.46	5.67 ± 1.67	69.63	5.67 ± 2.07	67.91	0.926
Post intervention 2	4.40 ± 2.04	59.74	4.40 ± 1.67	59.53	5.70 ± 2.03	84.72	< 0.001
*P *value for within groups	< 0.001		< 0.001		0.674		
Post-pre intervention 1	-0.725 ± 0.640	57.19	-0.659 ± 0.701	61.30	-0.089 ± 0.999	85.51	0.001
Post-pre intervention 2	-1.178 ± 0.824	54.83	-1.266 ± 0.821	50.88	-0.037 ± 0.929	98.29	< 0.001

 The three groups were compared in terms of breastfeeding frequency and duration in the first 18 hours of birth, as well as the mean birth weight of the infants and weight changes from birth until 10 days later. There was a significant difference across the groups regarding breastfeeding frequency and duration. There was no significant difference across the three groups regarding the mean birth weight; however, the mean weight changes between birth and the 10^th^ day differed in the three groups. In addition, the mean weight changes were negative in the control group but positive in the other two groups (Table 3). Nevertheless, a pairwise comparison revealed lack of a significant difference between the reflexology and the Benson relaxation groups in terms of breastfeeding frequency (*P* = 0.403) and duration (*P* = 0.750). Breastfeeding time (*P* < 0.001) and duration (*P* < 0.001) were significantly higher in the reflexology group, compared to the control group. Furthermore, breastfeeding time (*P* < 0.001) and duration (*P* < 0.001) were significantly higher in the Benson relaxation group, compared to the control group. In addition, a pairwise comparison showed that the mean weight changes of neonates were significantly higher in the reflexology (*P* = 0.004, 95% CI = 40.101‒202.121) and the Benson relaxation (*P* = 0.014, 95% CI = 20.723‒182.744) groups, compared to the control group. However, we found no significant difference between the reflexology and the Benson relaxation groups in this respect (*P* = 0.637, 95% CI = -61.633‒100.388).

**Table 3:** Comparisons of the Frequency and Duration of Breastfeeding During 18 Hours after Birth, as well as Mean Weight Changes from Birth Until the 10th Day Across the Three Groups

**Variables**	**Reflexology**		**Benson Relaxation**		**Control**		***P*** ** Value**
	**Mean±SD**	**Mean Rank**	**Mean±SD**	**Mean Rank**	**Mean±SD**	**Mean Rank**	
Number of breast feeding during 18 h after birth*	12.24 ± 2.42	86.52	11.64 ± 2.06	79.17	10.09 ± .79	38.31	< 0.001
Duration of breast feeding during 18 h after birth/ Minutes*	116.00 ± 41.15	82.87	117.33 ± 33.92	84.64	82.09 ± 13.38	36.49	< 0.001
Birth weight/gram**	3102.88 ± 422.68	-	3258.38 ± 445.58	-	3091.56 ± 340.17	-	0.097
Mean change between birth weight and tenth day weight/gram (10th day- birth day weight)**	111.55 ± 149.57	-	92.18 ± 268.30	-	-9.56 ± 137.31	-	(0.008)

* Performed statistical test is Kruskal-Wallis; ** Performed statistical test is One-way ANOVA.

 The results of the regression analysis in the first episode of the intervention showed that after adjusting for baseline pain, the post-intervention pain scores less than the median in the reflexology and the Benson groups were about 3.6 and 3.7 times more frequent than the control group, respectively. Regarding the second episode of the intervention, after adjusting for baseline pain, the post-intervention pain scores less than the median in the reflexology and the Benson groups were 2.9 and 4.7 times more frequent than the control group, respectively.

 After adjusting for the mother’s age, employment status, history of previous C/S and causes of C/S, the employment status, history of previous C/S and causes of C/S, the rate of return to birth weight in ten days from birth in the reflexology and the Benson groups were about 3.7 and 2.8 times higher than the control group, respectively.

 With regard to the frequency of breastfeeding during the first 18 hours after C/S, by adjusting for the mother’s employment status and the mean of all pain scores, it was revealed that the frequency of breastfeeding above 10 times (as median) in the reflexology and the Benson groups was about 6 and 4 times higher than the control group, respectively (Table 4).

**Table 4:** Regression Analysis Results – The Intervention Effect on Post-intervention Pain Less Than the Median, Frequency of Breastfeeding During 18 Hours Greater Than the Median and Return to the Birth Weight 10 Days from Birth

**Outcomes**	**Intervention**	**No. (%)**	**Adjusted OR**	***P*** ** Value**	**95% CI for OR**
Mean pain score after the first episode of the intervention below the median (vs. above the median)*: median = 6.6	Reflexology	27 (60) vs 18 (40)	3.569	0.007	1.419‒8.976
	Benson	29 (64.4) vs 16 (35.6)	3.692	0.006	1.451‒9.390
	Control	9 (20) vs 36 (80)	1	‒	‒
Mean pain score after the second episode of the intervention below the median (vs. above the median)**: median = 4.3	Reflexology	20 (44.4) vs 25 (55.6)	2.879		1.322‒6.272
	Benson	24 (53.3) vs 21 (46.7)	4.713		2.087‒10.644
	Control	12 (26.7) vs 33 (73.3)	1	‒	‒
Return to the birth weight ten days from birth (vs non return)***	Reflexology	36 (80) vs 9 (20)	3.730	.008	1.407‒9.886
	Benson	32 (71.1) vs 13 (28.9)	2.809	.031	1.100‒7.176
	Control	25 (55.6) vs 20 (44.4)	1	‒	‒
Proportion of breastfeeding more than the median (vs. less than median) during 18 hours ****: median = 10 time	Reflexology	38 (84.4) vs 7 (15.6)	6.077	0.001	2.026‒18.229
	Benson	35 (77.8) vs 10 (22.2)	4.124	0.008	1.443‒11.792
	Control	16 (35.6) vs 29 (64.4)	1	‒	‒

*OR adjusted for pain score before the first episode of the intervention. ** OR adjusted for pain score before the second episode of the intervention. *** OR adjusted for the mother’s age, employment status, history of previous C/S, causes of C/S. **** OR adjusted for the mother’s employment status, mean of all pain scores. N, Number; CI, confidence interval; C/S, Cesarean section.

## Discussion

 The present study aimed to compare the effect of the reflexology and the Benson relaxation methods on pain, breastfeeding, and infant weight gain in mothers undergoing a C/S. According to the results, the two intervention techniques had similar impacts on pain, breastfeeding frequency and duration, and infant weight gain. In addition, pain decreased similarly in the two intervention groups. The proportion of post-intervention pain less than the median, as well as the frequency of breastfeeding more than the median; and return to the birth weight ten days from birth were significantly higher in the two intervention groups than the control group. Consistent with our findings, previous studies have shown pain reduction in their samples.^5,13,31,32^ However, few studies have compared the two groups used in the current research. In two studies comparing foot reflexology and hand reflexology with Benson relaxation, reflexology had a higher impact on pain reduction,^23,25^ which is not consistent with our findings. This discrepancy might be due to the process of Benson relaxation, which is controlled by the participants. This difference could also be related to the training technique used with the participants. Reflexology alleviates pain by increasing the level of endorphin, which is a morphine-like substance.^5^ On the other hand, Benson relaxation reduces pain by decreasing tissue oxygen demand and the level of chemicals (e.g. lactic acid), as well as eliminating tension and stress in muscles and releasing endorphins.^33^ Given the similar effect of the two techniques, either can be used for pain relief depending on the available resources.

 According to the results of the present study, the frequency and duration of breastfeeding were similar in the Benson relaxation and the reflexology groups and higher than the control group. Our findings are in accordance with the results obtained by other studies in terms of the effect of reflexology on breastfeeding.^2,17,19^ In a research by Dhanalakshmi et al, there was a negative correlation between breastfeeding and pain, and breastfeeding increased following the implementation of the pain management intervention.^17^ In a study conducted in Saudi Arabia, Albokhary and James1 marked a higher possibility of formula feeding by women undergoing a C/S, compared to those who had a natural delivery. According to these studies, poor attachment to the neonate and pain were the causes of formula feeding.^1^

 In a study by Babazadeh et al, pain following a C/S disrupted breasting initiation.^34^ In the present study, the two reflexology and Benson relaxation methods improved breastfeeding to a similar extent. In addition, their similar impact on breastfeeding was expected given their similar effect on pain reduction. Nonetheless, the relationship between the researcher and the participants and her presence at their hospital beds (to carry out the intervention) might have affected breastfeeding frequency, especially in the reflexology group. However, this hypothesis is not strong considering the lack of difference between the reflexology group (which is an operator-dependent technique) and the Benson relaxation group (which is a client-oriented method).

 Furthermore, the results revealed that the difference between the infants’ 10-day weight and birth weight was positive in the two intervention groups and negative in the control group. Also, the rate of return to the birth weight ten days from birth was significantly higher in the two intervention groups than the control group. In the available literature, we found no research evaluating the effect of complementary and alternative therapies on the infant’s weight gain in addition to their effect on pain. According to the study hypotheses, the infants’ weight gain during the first 10 days after birth was due to better breastfeeding by the participants who had experienced less pain; and this could be explained based on studies that have also evaluated the relationship between breastfeeding and infants’ weight reduction or weight gain. Davanzo et al found that C/S or the use of formula were associated with the loss of ≥ 8% of weight during the hospital stay.^35^ Thulier reported a high decline in exclusive breastfeeding for neonates experiencing weight loss of > 7%.^36^ In another study, the rate of return to birth weight on the 14^th^ day was lower in neonates born via C/S, compared to those born through natural delivery.^37^ In C/S, the hormone pathway that stimulates lactogenesis is disrupted by maternal stress or reduced oxytocin secretion, and can inhibit milk production.^38^ In addition to pain, analgesics and opioids decreased breastfeeding.^37^

 One of the limitations of the present study was the inability to find similar studies assessing the effect of reflexology and relaxation on infants’ weight gain (on websites available to the researcher). Therefore, we determined the sample size using studies that evaluated the effect of the intervention on pain and breastfeeding. Moreover, maternal and neonatal states during the first 10 days of birth might have affected breastfeeding and infant weight gain. Some factors, including the social support received by the mother, mild postoperative complications at home (e.g. infection and inflammation of the surgical site), and mild neonatal diseases (e.g. gastroenteritis) which do not need hospitalization were not considered in the present study, and can be assessed in future research.

 In conclusion,both the reflexology and the Benson relaxation methods decreased pain and affected breastfeeding frequency and infant weight gain following a C/S. We found that both the reflexology and the Benson relaxation interventions had relatively similar impacts. Thus, it is recommended to employ these interventions as a complementary or alternative method (based on patients’ preferences and appropriate performance conditions) to decrease post-C/S pain and enhance breastfeeding at birth and infant weight gain.
